# Early Resistance Rehabilitation Improves Functional Regeneration Following Segmental Bone Defect Injury

**DOI:** 10.21203/rs.3.rs-3236150/v1

**Published:** 2023-10-04

**Authors:** Kylie E. Williams, Julia Andraca Harrer, Steven A. LaBelle, Kelly Leguineche, Jarred Kaiser, Salil Karipott, Angela Lin, Alyssa Vongphachanh, Travis Fulton, J. Walker Rosenthal, Farhan Muhib, Keat Ghee Ong, Jeffrey A. Weiss, Nick J. Willett, Robert E. Guldberg

**Affiliations:** 1 Phil and Penny Knight Campus for Accelerating Scientific Impact Department of Bioengineering, University of Oregon, Eugene, OR 97403; 2 Wallace H. Coulter Department of Biomedical Engineering, Georgia Institute of Technology and Emory University, Atlanta, GA; 3 Atlanta Veteran’s Affairs Medical Center, Decatur, GA; 4 Emory University, Decatur, GA; 5 Department of Biomedical Engineering, University of Utah, Salt Lake City, UT 841123; 6 Department of Orthopaedics, University of Utah, Salt Lake City, UT 841123; 7 Scientific Computing and Imaging Institute, University of Utah, Salt Lake City, UT 84112

## Abstract

Mechanical loading is integral to bone development and repair. The application of mechanical loads through rehabilitation are regularly prescribed as a clinical aide following severe bone injuries. However, current rehabilitation regimens typically involve long periods of non-loading and rely on subjective patient feedback, leading to muscle atrophy and soft tissue fibrosis. While many pre-clinical studies have focused on unloading, ambulatory loading, or direct mechanical compression, rehabilitation intensity and its impact on the local strain environment and subsequent bone healing have largely not been investigated. This study combines implantable strain sensors and subject-specific finite element models in a pre-clinical rodent model with a defect size on the cusp of critically-sized. Animals were enrolled in either high or low intensity rehabilitation one week post injury to investigate how rehabilitation intensity affects the local mechanical environment and subsequent functional bone regeneration. The high intensity rehabilitation animals were given free access to running wheels with resistance, which increased local strains within the regenerative niche by an average of 44% compared to the low intensity (no-resistance) group. Finite element modeling demonstrated that resistance rehabilitation significantly increased compressive strain by a factor of 2.0 at week 1 and 4.45 after 4 weeks of rehabilitation. The resistance rehabilitation group had significantly increased regenerated bone volume and higher bone bridging rates than its sedentary counterpart (bone volume: 22.00 mm^3^ ± 4.26 resistance rehabilitation vs 8.00 mm^3^ ± 2.27 sedentary; bridging rates: 90% resistance rehabilitation vs 50% sedentary). In addition, animals that underwent resistance running had femurs with improved mechanical properties compared to those left in sedentary conditions, with failure torque and torsional stiffness values matching their contralateral, intact femurs (stiffness: 0.036 Nm/deg ± 0.006 resistance rehabilitation vs 0.008 Nm/deg ± 0.006 sedentary). Running on a wheel with no resistance rehabilitation also increased bridging rates (100% no resistance rehabilitation vs 50% sedentary). Analysis of bone volume and von Frey suggest no-resistance rehabilitation may improve bone regeneration and hindlimb functionality. These results demonstrate the potential for early resistance rehabilitation as a rehabilitation regimen to improve bone regeneration and functional recovery.

## Introduction

Non-union fractures present a significant clinical challenge, requiring surgical intervention and often result in post-operative complications. While most bone fractures heal spontaneously without surgical or therapeutic intervention, up to 5–10% of fractures fail to heal and result in non-union^1^. Non-union rates are highest in severe bone fractures including open fractures, injuries with critical bone loss or damage to surrounding tissue, and in patients with co-morbidities. These types of fractures are often treated with surgical reconstruction and stabilization with fixation hardware in combination with bone grafts or other regenerative treatments as needed^2^. The complex nature of severe bone injuries often results in post-operative complications due to infection, hardware failure or misplacement, or lack of regenerative response, which can lead to costly subsequent surgical and therapeutic intervention^3^. In addition to surgical repair, patients are often prescribed rehabilitation to promote functional recovery. However, rehabilitation regimens are often conservative and begin with extended periods of non-loading for up to 12 weeks. Patients are then allowed to progress through partial weight bearing, range of motion and muscle strengthening exercises, and full weight bearing before eventual return to function^4,5^. Success of these rehabilitation regimens also relies on patients’ discipline, communication, and judgment of pain and swelling^6^. After this extended process, patients are often left with extensive atrophy and fibrosis of the surrounding muscle and soft tissue, even in cases that are considered successful surgical management and bone repair^7–9^. In the case of severe injuries, patients may require multiple surgeries and years of painful recovery, leaving them with long-term disability and/or amputation due to unmanageable complications^2,10^. These factors underscore the need for improved rehabilitation strategies that strive for consistent functional recovery of bone and surrounding tissue.

The emerging field of regenerative rehabilitation focuses on early integration of rehabilitation to enhance the body’s capacity to restore function to the injured limb. It is well established that musculoskeletal cells and tissues (including bone) respond and adapt to mechanical stimuli^11–13^. During bone regeneration and fracture healing, mechanobiology plays a pivotal role dictating the type of healing process through a relationship between fixation, tissue-level strains, and interfragmentary movement. Fixation strategies that load the fracture site lead to callus formation and endochondral ossification, resulting in increased cartilage and chondrocytes in the bone regenerative niche. This supports the hypothesis that increased interfragmentary movement promotes endochondral ossification, whereas stable fixation promotes intramembranous ossification by shielding the fracture from strains^14–16^. However, fixation strategies resulting in excessive strain are detrimental to healing and result in non-union^17,18^. In the clinic, physical stimuli can modulate biochemical signaling pathways to stimulate the regenerative response of the target tissues^19^. Using this concept, researchers are leveraging mechanical stimuli for functional healing of weight-bearing musculoskeletal tissue.

Rehabilitation strategies for bone healing require a careful balance between the stability of the bone fixation system and the initiation of mechanical stimulation. Pre-clinical research has begun interrogating this balance through the modulation of fixation plate designs and subject activity. However, lack of standardization between studies and models have led to conflicting results. Several studies have documented that low fixation stiffness is detrimental to healing, as it allows for excessive strain and interfragmentary motion^14,20,21^. Meanwhile, other studies have observed that increased fixation stiffness suppressed callus formation and subsequent bone healing compared to compliant fixation systems^15,22–25^. Researchers have also investigated how the onset of increased mechanical stimuli affects bone healing. Studies have demonstrated that reducing plate stiffness (i.e. dynamization) at 1 week post injury did not improve bone healing and impeded vascularization into the defect region^16,26^. Conversely, another study showed that dynamization at 1 or 2 weeks post injury led to improved regenerated bone volume and mechanical properties overall, but the course of helaing occurred at a slower rate^27^. Similar trends were seen when dynamization began at 3–4 weeks post injury^16,21,28,29^. More recently, it was found that increasing plate stiffness after the initial callus formation (i.e. reverse dynamization) accelerated bone healing and mechanical strength^14,24,30,31^. Specifically, these studies found that increased mechanical stimulation promoted soft callus formation during the early phases of healing, while rigid fixation promoted mineralization in the later stages of bone healing^30^. This lack of consensus regarding the timing and magnitude of mechanical stimulation is largely due to the lack of standardization between studies regarding the methods and magnitude of mechanical stimulation, fixator stiffness, bone defect size, and animal model^32–34^. Regardless, the principles of mechanobiology suggest that healing responses depend on tissue-level strains. Thus, discrepancies in the literature may be resolved by investigating how individual rehabilitation strategies alter the transfer of ambulatory strains from fixation plates to the regenerative niche. Further, this approach could identify optimal rehabilitation parameters to enhance healing and functional recovery.

Recently, implantable mechanical load sensors were developed to measure the mechanical stimuli experienced in implants and regenerating tissue. Windolf et al. developed an implantable load sensor that continuously monitored implant loading over several months of fracture healing^35^. The implantable sensor enabled the evaluation of bone healing in a sheep mid-shaft tibial osteotomy model and could eventually be utilized to evaluate patient healing and develop patient-specific treatment strategies^36^. Barcik et al. also employed a force sensor in sheep to measure the stiffness of the regenerated tissue. The fixator enabled this study to implement loading protocols that were modified based on healing progression^37^. These technologies have the potential to provide real time, continuous insight into the relationship between loading parameters and bone healing. However, target load parameters and load histories to stimulate functional bone formation have not yet been established.

Previously, our labs developed implantable, wireless strain sensors and coupled them with finite element models to longitudinally calculate the tissue-level strains following injury and rehabilitation^25^. In a study comparing stiff vs compliant fixation plates, we found that the compliant fixation group demonstrated improved mineralized bridging, 60% greater bone formation, and a two-fold increase in strain magnitude compared to the stiff fixation group^25^. However, torsional stiffness of regenerated femurs from either group did not reach that of intact, contralateral limbs, indicating the potential for further modulation to the local mechanical environment to improve functional healing^25^. Furthermore, defects were supplemented with bone morphogenetic protein 2 (BMP-2), which is rarely used for extremity trauma management in the clinic, limiting the generalizability of these results^38^. Beyond modulating fixation plate materials and design, the mechanical loads and tissue strains can be altered through rehabilitation intensity. Other musculoskeletal injury models have found beneficial effects of increasing exercise intensity, however full characterization of rehabilitation parameters is still lacking in the field^12^.

In this study, we aimed to elucidate the effects of strain within the regenerative niche on functional segmental bone healing in a rat model. To do so, we surgically created subcritical (2 mm) defects, allowed animals to recovery in sedentary conditions for 1 week, and then animals were either kept in sedentary conditions for the remainder of the study or randomly allocated to rehabilitation with a running wheel with (resistance rehab) or without (no resistance rehab) resistance that was applied by a programmable brake. The impact of rehabilitation on the local mechanical environment and subsequent functional bone healing were longitudinally monitored through a series of studies by using custom implantable strain sensors, radiographs, and microCT scans. Mechanical testing and histology of explant femurs were performed to investigate how the rehabilitation intensity impacted bone healing. Finally, subject-specific finite element simulations were performed to measure the strains within the regenerative niche based on longitudinal strain data from fixation plate sensors. We hypothesized that mechanical loads induced by early resistance rehabilitation would result in increased local strains within the regenerative niche that would then accelerate bone formation and improve functional recovery. This approach may ultimately advance the field of regenerative rehabilitation by enabling data-informed rehabilitation decisions to improve functional recovery following complex bone injuries.

## Methods:

### Strain Sensor Fabrication

A digital transceiver unit was developed as previously described^39^. This device relies on wireless Bluetooth Low-Energy (BLE) microcontroller (MCU) to receive commands and transmit data via a custom PC software (Visual Studio) and is powered by a 620 mAh lithium coin-cell (CR2450, Panasonic). The transceiver unit was encased in a 3D printed housing unit with two multistranded stainless steel wires (A-M systems) connected to a strain sensor (EA-06–125BZ-350/E, Micro-measurements) integrated into a custom internal fixation plate used to stabilize the femoral defect. The internal fixation plate is radiolucent and fabricated with ultra-high-molecular-weight polyethylene (UHMWPE, Quadrant) to promote load sharing across the defect. Before implantation, each device was calibrated using a three-point bending flexural test on a tensile testing machine (TA Electroforce 3220) to physiologically relevant strain magnitudes^39^.

### Surgical Procedure

As previously described^15,25,38–41^, unilateral 2 mm segmental defects were created in the left femur of 17-wk old female SASCO rats (Sprague Dawley, Charles River Labs). Femurs were stabilized with load-sharing internal fixation plates prior to creating the defects^25^. A subset of animals within each group were provided fixation plates integrated with strain sensors. For these animals, transceiver packs were mounted in the abdominal cavity and the wire connecting these pieces were subcutaneously fed through a keyhole incision in the abdominal wall superior to the left inguinal ligament. Defects were left empty to better discern the impact of exercise without biologic treatment as a confounding factor. Animals were randomly allocated to experimental groups: sedentary control, no resistance rehabilitation, or resistance rehabilitation. Animals were euthanized by CO_2_ asphyxiation after 8 weeks of recovery. All procedures were approved by the University of Oregon IACUC or Atlanta Veteran’s Affairs Medical Center IACUC.

### Voluntary Wheel Running

Prior to surgery, rats were acclimated to running wheels where they had voluntary access to in-cage running wheels (Scurry Rat Running Wheel, Lafayette Instruments^®^). After surgery, rats were singly housed and allowed 7 days to recover. Non-sedentary animals were then granted voluntary access to a running wheel with either no resistance or pre-programmed 25%–40% body weight resistance applied via programmable brakes (rat brake, Lafayette Instruments^®^). Running wheels with resistance created higher intensity rehabilitation due to the friction between the resistance brake pad requiring greater force to move the wheel (Fig. 1a). Animals were weighed on a weekly basis and the resistance levels were changed to ensure resistance levels were maintained throughout the 7 weeks of rehabilitation. Running activity (m/day) was longitudinally monitored using onboard rotation monitoring sensors (Scurry Rat Activity Sensor, Lafayette Instruments^®^). Animals in the sedentary group were singly housed in standard cages with no wheel for the full 8-week recovery duration.

### Wireless Strain Data Acquisition and Analysis

Strain measurements were transmitted in real-time via Bluetooth connection to a nearby laptop and plotted on a custom Microsoft Visual Studio C# graphical user interface (GUI) during 10-minute wheel running sessions performed twice weekly. These measurements started after the 7-day post injury recovery period and continued until the week 8 endpoint. Strain measurements were collected for animals with implanted sensors and given access to a wheel (no resistance and resistance animals). A custom MATLAB (Mathworks) script identified local maxima and minima pairs to track individual step cycles and compute the average 90^th^ percentile of strain amplitudes per animal across each week of post injury rehabilitation.

### Gait Analysis

Post injury hindlimb function was assessed using an Experimental Dynamic Gait Arena for Rodents (EDGAR) that provided quantitative assessment of rodent hindlimb function^42^. Prior to surgery, rats were acclimated to the gait arena for 20 mins across 3 consecutive days. Gait was assessed at baseline (1 week prior to surgery) and at weeks 1, 3, and 8 post injury with a high-speed camera (500 fps, 1920×1200, Phantom Micro 320, Vision Research, Inc, Wayne, NJ). Videos were processed in the EDGAR software package^43^ which automatically tracked rats, isolated fore and hind limbs, identified foot-strike and toe-off, and calculated the following gait parameters: velocity, stance times, stride times, imbalance, step widths, stride length, and spatial/temporal symmetries (Supplemental Fig. S1). Spatiotemporal parameters were corrected for each rat’s mass and velocity based on a healthy databased established within our lab. Parameters are then reported as residuals from the expected value of a healthy rat at a given mass and velocity, as established by Kloefkorn et al.^42^.

### Von Frey Pain Testing

Mechanical allodynia of the hindlimbs was measured using a 50% withdrawal threshold with von Frey filaments. Animals were acclimated to the protocol with 3 days of consecutive pre-testing. Von Frey filaments (Stoelting, Wood Dale, IL, USA) were applied through the bottom of a wire cage and held to the plantar surface of the hind paws for 5s. A positive response was defined as the animal withdrawing, licking, or shaking its paw. The protocol started with a moderate filament (10 gram-force) and then moved to a smaller filament if there was a response or a larger filament if there was no response. Only one stimulus was applied per minute until 5 total stimuli were applied, or until a threshold of 26 gram-force was reached.

### Ex vivo microCT and biomechanical testing

Animals were euthanized by CO_2_ asphyxiation, left and right femurs were dissected and cleaned of soft tissue, fixation plates were carefully removed from the left femurs, and femurs were wrapped in PBS-soaked gauze and placed in 15mL conical tubes at 8 weeks post injury. Ex vivo microCT scans were performed by centering the conical tubes on the appropriate tube holder (VivaCT 80, Scanco Medical) and using 36 μm voxel, 70 kVp, 114 μA, and 200 ms integration time for the initial pilot study or 24 μm voxel, 55 kVp, 145 μA, and 750 ms integration time for the follow-up studies. After scans, all femurs were stored at −20 °C in phosphate buffered saline (PBS) soaked gauze until biomechanical testing was performed 3 days later. As described in Klosterhoff et al., explanted left (defect) and right (intact) femurs were biomechanically tested to determine the failure strength and torsional stiffness^25^. Ex vivo femurs were tested to failure in torsion at 3°/sec using a load frame (TA Electroforce 3220). Functional analysis involved inter-animal comparisons between defect (left) and intact (right) femurs as well as comparisons between the sedentary and resistance rehabilitation groups.

### Finite Element Analysis

The mechanical environment (compressive and shear strain) in the rehabilitative niche was quantified by finite element simulations of one no resistance- and one resistance-running animal at 1- and 4-weeks of post injury rehabilitation. First, an idealized geometric assembly of a left femur and fixation plate assembly was created from our previously published study^25^. Geometries for week one of post injury rehabilitation models were created by sectioning the intact idealized femur to create a 2.0 mm defect. The top and bottom of the defect were treated as fibrous ectopic tissue. The middle region of the defect comprising the granulation tissue was modeled by generating a cylinder between the proximal and distal ends of the femur. Geometries for week four of post injury rehabilitation models were similarly derived from the idealized initial geometry. Subject-specific defect geometries were extracted from microCT images and then the idealized femur was aligned with the visible portions of intact bone in the defect microCT images. Ectopic bone surfaces were approximated from radiographic measurements in the current study. All geometries were meshed with 10-node quadratic tetrahedral elements of a similar size as used in our previous models, which were validated by mesh convergence studies^25^. Nodes between model components were welded into a single compatible mesh such that contact did not need to be explicitly modeled. Geometries, volume meshes, and material property assignments were performed in Mimics and 3-Matic (Materialise). Simulations were performed in FEBio (www.febio.org, version 3.7, Salt Lake City, UT) and results were visualized with FEBioStudio (version 2.0)^44^.

All materials were modeled as isotropic elastic solids. The material coefficients for the sensor plate, riser, and intact femur were assigned from our previously published study^25^. The same homogenous material properties were assigned regardless of rehabilitation intervention since minimal healing and tissue differentiation were expected at 1-week post injury. Granulation tissue and fibrous tissues were assigned material coefficients from the literature (Supplemental Fig. S2,^45^). Material coefficients for the defects at 4-weeks post injury were derived from microCT image data. The Young’s modulus was assigned to each finite element from the relationship $E=s_{E}\rho^{1.49}$ where *s*_*E*_ is a scalar (MPa) and *ρ* is the local image intensity (arbitrary units in the range [−1000, 12631]). The Poisson’s ratio was assigned to each finite element from the relationship $v=s_{v}\rho^{0.64}+0.166$ where *s*_*v*_ is a scale factor (no units). These coefficients were selected so that the values agreed with prior literature (Supplemental Figs. S2, S3,^25,45^). Ectopic growth was not entirely detected by the microCT images; thus, ectopic tissues were assumed to be homogenous with material coefficients derived from the literature^46^.

Model loading and boundary conditions were prescribed similar to previous simulations (Supplemental Fig. S3,^25,47^). The distal end of the femur was fixed in all directions. A spatially-varying pressure was assigned to facets on the surface of the femoral proximal surface from the Euler-Bernoulli beam-bending relationship:

(1) $$\sigma =\sigma_{c}+\sigma_{ml}+\sigma_{ap}=A\left(\frac{1+0.1202y-0.2404x}{\;\text{total}\;\text{cortical}\;\text{area}\;}\right)$$

The total normal stress *σ* was the sum of the axial compressive stress (*σ*_*c*_), the bending stress about the medial-lateral axis (*σ*_*ml*_), and the bending stress about the anterior-posterior axis (*σ*_*ap*_). Here, *x* was the medial position of the element surface and *y* was the anterior position of the element surface. The parameter *A* was iteratively varied until the average compressive (3^rd^ principal Lagrange) strain across the fixation plate matched experimental measurements of compressive strain from the implantable strain sensors for each model (Supplemental Figs. S4, S5). Compressive (3^rd^ principal) and shear strain values were then extracted from the elements in the defect to generate descriptive statistics and comparative analyses between models. Elements from mineralized tissues were excluded to restrict the analysis to the granulation tissue.

### Statistical Analysis

All statistical analyses were performed with Prism 9 (GraphPad) unless otherwise noted. Ordinary One-Way ANOVA with Tukey’s multiple comparisons was used to compare endpoint bone volume across experimental groups. Mixed effect analyses were used to investigate which variables (time, rehabilitation condition, or the interaction) may contribute to difference in functional outcomes differences between sedentary and no resistance animals. Average weekly distance (m/day) across time and between rehabilitation groups were assessed by multiple unpaired t-tests. Multiple t-tests with multiple comparisons and Holm-Šídák corrections were also utilized to investigate the mechanical properties of explanted femurs from animals in sedentary conditions versus resistance rehabilitation. Data are displayed as mean ± SEM since experimental groups had unequal sample sizes due to postoperative complications and the inclusion of multiple studies. Mann-Whitney U-tests were performed for measurements derived from FE simulations using Origin 2020b (OriginLab, Northampton, MA). Statistical significance is shown with asterisks and exact p-values are listed in figure headings.

## Results:

### Early resistance rehabilitation improved bone healing

The effect of rehabilitation intensity on bone healing was assessed using a femoral segmental 2 mm bone defect model, which is on the cusp of a critically-sized bone defect. Bone regeneration was assessed longitudinally by radiographs and microCT. After 8 weeks of recovery, 50% of sedentary animal femurs experienced bridging compared to 90% of rehabilitated animal femurs, suggesting that a 2 mm segmental bone defect in female Sprague Dawley rats is not consistently critically-sized (Supplemental Fig. S6). Early rehabilitation was associated with significantly greater bone volume within the defect region of microCT scans when compared to sedentary counterparts (Fig. 1b). In addition, early (4 week) callus formation and mature callus consolidation (week 8) were only observed in radiographs of resistance rehabilitation animals (Fig. 1c). Although the majority of femurs from sedentary and no resistance animals were bridged by week 8, radiographs and microCT reconstructions revealed distinguishably less dense tissue compared to intact bone throughout the defect region, suggesting less mature healing (Fig. 1c, d). Taken together, early resistance rehabilitation was shown to improve bone formation which may be explained by higher intensity exercise leading to early callus formation and more mature healing within our 8-week timeframe.

Histological analysis at week 8 suggests different mechanisms of bone formation across groups (Fig. 2). In sedentary animals, appositional bone formation extends from both ends of the bone with fibrous tissue in the center, while the resistance animals displayed a robust callus bridging the defect site (Fig. 2a,b). Fibrous tissue was visible in the center of the defect in the sedentary rats, while both cartilage and woven bone were visible in the callus of the resistance group (Fig. 2c,d). This suggested that this bone regenerated through endochondral ossification (Fig. 2c,d). This is corroborated by other data demonstrating that lower strains correspond to appositional bone formation, while greater strains in the rehabilitation group promoted endochondral ossification. This indicates that promoting endochondral ossification by increasing local strains is beneficial to bone healing in defects on the margin of critically-sized.

To investigate the impact of resistance rehabilitation on the mechanical properties of the regenerated tissue compared to sedentary counterparts, we performed torsion testing to failure of the defect and intact femurs. Torsional mechanical testing revealed that resistance rehabilitation increased failure torque and torsional stiffness of defect limbs compared to those from sedentary animals (Fig. 3a, 3b). Further, resistance rehabilitation led to tissue healing with mechanical properties that matched intact femurs (Fig. 3a, 3b). Interestingly, the mechanical properties of intact femurs were significantly greater for animals that underwent resistance rehabilitation compared to intact femurs from sedentary counterparts (Fig. 3a, 3b), suggesting bone remodeling in response to seven weeks of resistance rehabilitation. Data were fitted to a linear mixed effects model which revealed a significant effect of surgery (p-value = 0.0491) and rehabilitation condition (p-value = 0.0034) on the endpoint failure torque of the femurs. However, the torsional stiffness of femurs was only significantly impacted by exercise conditions (p-value = 0.0050). Polar moment of inertia of the defect region and bone ends was also significantly increased for rats that underwent resistance rehabilitation compared to sedentary counterparts (Fig. 3c). These results were consistent with the greater callus formation observed in our histology and radiography. Taken together, resistance rehabilitation led to improved healing and functional recovery of femurs compared to sedentary animals.

### Early rehabilitation may improve pain and limb function

Several rehabilitation parameters may affect functional bone healing outcomes, including rehabilitation intensity and volume. In these studies, we prioritized assessing the effect of intensity by providing animals with unrestricted access to a running wheel with or without resistance applied. Activity level (m/day) could have been a confounding variable for healing outcomes since animals were given voluntary access to running wheels. However, we found no significant difference (p-value = 0.2131) in average weekly activity between the no resistance and resistance rehabilitation groups, indicating that the differences observed in healing were likely not due to the amount of activity (Fig. 4a). Both groups decreased activity from pretraining (3297 m/day ± 471.1, week −1) to week 2 (1802 m/day ± 367.2) following surgery (Fig. 4a). Activity then increased to post operative levels by week 3 and continued to increase until week 5, at which point activity plateaued between 4500–5500 m/day (Fig. 4a).

In addition to bone healing and function, rehabilitation has the potential to improve gait and mechanical allodynia. A subset of the sedentary (n = 4) and no resistance rehabilitation (n = 3) groups were evaluated for pain sensitivity using von Frey filaments to measure mechanical allodynia and for spontaneous gait using an Experimental Dynamic Gait Arena for Rodents (EDGAR). In the von Frey filament analysis, a mixed effects model showed a significant decrease in the overall withdrawal response week 3 compared to baseline, indicating allodynic pain (p-value = 0.0018); no difference between the treatment groups was observed at week 3 (Fig. 4b). At week 8, the no resistance rehabilitation animals had a significant increase in the withdrawal response compared to the sedentary animals (p-value = 0.0224), indicating pain relief. Gait analysis showed an initial functional deficit in response to the segmental bone defect injury (Supplemental Fig. S1). Using a mixed effects model, there was a significant decrease in the overall left hind duty factor, hind duty factor imbalance, and hind spatial symmetry 1 week after injury, indicating a decrease in the time the left limb spent on the ground and asymmetric placement of hind limbs after injury. No significant differences were observed between the sedentary and rehabilitation treatments for any gait parameters.

### FEA revealed resistance rehabilitation increased compressive and shear strain within the regenerative niche

Physical rehabilitation is a promising approach to mechanically stimulate the regenerative niche and encourage the body’s endogenous capacity to heal. Variability in rehabilitation regimens as well as the dependence of tissue differentiation on load onset and magnitude makes it difficult to establish a consensus from the existing literature. Therefore, we assessed the temporal progression of mechanical cues by measuring internal bone plate strain during running wheel activity starting one week after surgery. Further, *in vivo* ambulatory strain measurements were monitored in a subject-specific, real-time manner by our previously developed and validated strain sensor integrated that we integrated into the internal fixation plates^39^. Dynamic strain cycle amplitudes corresponding to individual steps were computed from strain sensor measurements and the 90^th^ percentile strain magnitudes were recorded for 7 weeks of post injury rehabilitative running (Fig. 5c). Ambulatory strains across the fixation plate gradually declined until bridging was observed in radiographs and microCT scans (Fig. 5c), which is consistent with previous literature^25^. FE models were then developed using ambulatory strains and microCT images to predict the defect level strains for each physical rehabilitation regimen at 1 or 4 weeks of rehabilitation (Fig. 5a). On average, resistance rehabilitation was associated with a 2.2 fold increase in plate compression throughout week 1 of rehabilitation (Table 1). FE simulations indicated a significant, 2.0-fold increase in 3^rd^ principal strain within the defect region due to resistance (p < 0.001; Fig. 5d,e). For these simulations, compressive strain was greatest in the region spanning the proximal-medial and distal-lateral ends of the defect. Similar trends were observed for shear strain (p < 0.001: Supplemental Fig. S7). Throughout week 4 of rehabilitation, resistance was associated with an 3.3-fold increase in plate compressive strain and a significant, 4.45-fold in defect compressive strain due to resistance (p < 0.001; Fig. 5f,g). Here, compressive strain was greatest on the lateral ends of the bones near the interfaces between intact femurs and defect tissues. Again, similar trends were observed for shear strain in response to resistance rehabilitation (p-value < 0.001; Supplemental Fig. S7). To approximate the changes in strain transfer between the fixation plates and the healing defects, we measured the ratio of the average compressive strain in the defect to measured compressive strain on the plate surface. There was a drastic reduction in this metric between weeks 1 and 4 for both animals (over 8-fold for the no-resistance subject, over 6-fold for the resistance running subject) due to bone formation in defects. Notably, defect/plate strain ratio was greater in the no resistance running animal at week 1, but the defect/plate strain ratio was greater in the resistance running animal at week 4.

## Discussion:

The benefits of mechanical stimulation for musculoskeletal repair have been investigated using strategies such as axial loading via flexible fracture fixation^25,28,48^ and controlled mechanical loaders^49^, low-frequency vibration treatment^50^, and ultrasonic stimulation^51,52^. However, there remains a clinical need for data-informed rehabilitation regimens, and a better understanding of how principles of mechanobiology are translated into clinical decisions that improve functional outcomes. To address this need, we investigated the impact of running intensity during early rehabilitation on healing outcomes and tissue biomechanics throughout the course of bone healing. Femurs with segmental defects were stabilized with compliant fixation plates integrated with sensors that measured local axial strain during low (no resistance) versus high (resistance) intensity running. Bone healing was monitored via longitudinal radiographs, microCT scans, as well as endpoint mechanical testing and histology. This study provides critical insight into the effects of rehabilitation intensity on bone healing after a segmental bone defect.

We study sought to assess functional bone healing after implementing a more rigorous rehabilitation protocol compared with our previous work that found improved bone formation with treadmill walking in combination with a low dose of local BMP-2 growth factor therapy^25^. In the present study, we utilized a smaller defect so the bone healing results was independent of exogenous growth factor dosing. Animals were provided voluntary access to running wheels, facilitating daily activity (m/day) that were at least 10x greater than treadmill running regimens^25^. Further, we utilized resistance brakes to modulate rehabilitation intensity. Early resistance rehabilitation not only induced significant improvement in bone formation and recovery of mechanical properties compared to sedentary counterparts, but preliminary results suggested early rehabilitation improved tactile pain response. Resistance rehabilitation with compliant fixation significantly increased the failure torque and torsional stiffness of both the defect and intact limbs as compared to sedentary counterparts. These data suggest that early resistance rehabilitation results in bone adaptation in the non-injured limb to withstand greater loads induced by resistance running, which is consistent with previous literature^12,53–56^. After segmental defect surgery, early resistance rehabilitation led to increased bone healing and strength and promoted full restoration of mechanical properties, which had not been previously achieved in our compliant fixation model. Unsurprisingly, implantable strain sensors measured an increase in fixation plate strain for resistance running animals compared to no resistance animals. Subject-specific finite element models revealed that resistance running induced a beneficial increase in compressive and shear strains within the regenerative niche as compared to no resistance running throughout weeks one and four of rehabilitation (Fig. 5, Supplemental Fig. S7). Overall, our data support early resistance rehabilitation as a promising therapeutic to increase bone formation, bone healing strength, and promote full restoration of mechanical properties to intact levels for defects on the margin of critically sized without the use of biologics.

The increased compressive and shear strains associated with resistance rehabilitation had a beneficial effect on healing of near critically-sized bone injuries; however, excessive strain can be deleterious. Boerckel et al. quantified the effect of early and delayed functional loading on neovascular growth in a rat model of 8 mm bone defect regeneration using compliant fixation plates^16^. In their study, fixation plates were unlocked to allow transfer of ambulatory loads to the defect either at the time of implantation or after 4 weeks of stiff fixation^16^. Early mechanical loading significantly reduced bone formation by 75% compared to stiff plate controls, which suggested that early loading of high magnitudes in a large defect is deleterious to healing^16^. Previous work by Ruehle et al. also found that immediate loading inhibited neovessel sprout tip cell selection and angiogenesis while delayed loading (five days) enhanced vascularization, suggesting the need for a brief, critical period of recovery prior to loading^57^. In acknowledgement of this necessary period of delay, we initiated resistance rehabilitation one-week post operation, resulting in enhanced bone healing of 2 mm segmental defects. However, the variable bone healing results across the literature highlight the significant need for personalized platforms to assess the impact of ambulatory loads on the local mechanical environment throughout healing.

Several studies have investigated how ambulatory load transfers allowed via compliant fixation plates impact healing of critically-sized bone defects treated with BMP-2^16,24,25,28,58^. Boerckel, et al. compared healing of 6mm defects stabilized with either a stiff fixation plate or a compliant plate and treated with 5 micrograms BMP-2. They found that delaying ambulatory loading until week 4 increased bone formation but did not result in mechanical restoration to intact levels. Similarly, Klosterhoff, et al. compared healing of 6mm defects treated with 2 micrograms BMP-2 and stabilized by either a stiff or compliant fixation plate^25^. Animals were also granted 20 minutes of treadmill walking per week starting after one week of recovery. Compliant fixation and treadmill walking resulted in increased compressive strain magnitudes, bridging rates, and bone formation, however, similarly did not achieve mechanical restoration^25^. Glatt et al., also compared healing of 5mm defects treated with 11 micrograms of BMP-2 and stabilized with external fixation plates at different stiffnesses^58^. Radiographs and histology revealed that low stiffness fixation produced the best healing after eight weeks^58^. A follow-up study then assessed healing due to constant fixation stiffness or reverse dynamization (fixation stiffness was increased after 2 weeks). MicroCT, histology, and radiographs revealed that reverse dynamization resulted in greater bone formation and mechanical properties that matched intact levels. All these studies found promising bone healing results with different loading regimens and timing, suggesting that bone healing response is more complex than merely a single strain value threshold. In addition, these studies did not result in complete functional restoration. It is also worth noting that these studies all supplemented rehabilitation with BMP-2, which has been established as a robust bone forming growth factor,^38,40,41,59,60^ and is likely a confounding factor in these healing results. In comparison, we found that resistance running significantly increase bone formation as compared to sedentary counterparts and restored femurs to intact strength without the use of biologics.

Although mechanical loading has been a longstanding therapeutic for musculoskeletal injuries, the magnitude of loading through all stages of fracture healing is not well understood^54,61,62^, largely because of the technological challenges of acquiring accurate, local measurements. We addressed this hurdle by utilizing implantable strain sensors to track longitudinal, real-time measurements of bone plate strain throughout rehabilitative activities to assess healing and predict bone healing well before radiographic evidence. In fact, the relationship between early strain amplitudes and healing outcomes previously revealed a significant positive relationship between strain amplitude one week after injury and regenerated bone volume after 8 weeks of healing^25^. For the present study, these same sensors revealed that resistance running increased local strains across the fixation plate and within the regenerative niche, which led to improved functional bone healing of 2 mm bone defects. A limitation of the sensor platform used for these rodent studies is that it requires a transceiver pack and has a restricted battery life. Further development of battery-free sensors that do not require a transceiver could facilitate clinical translation and potentially enable data-informed revisions to rehabilitation parameters that improve functional healing.

The goal of this study was to explore how rehabilitation intensity can be utilized to produce mechanical loading advantageous to bone healing of defects on the margin of critically-sized. We previously used finite element simulation to predict the difference in defect-level mechanics facilitated by stiff or compliant fixation plates supporting 6mm defects during two weekly, 10-minute treadmill walking at 3 weeks post injury^25^. Defects supported by compliant fixation plates experienced a 60% increase in bone formation when compared to defects supported by stiff fixation plates. Compliant plates facilitated defect compression between ~1–6% while stiff plates facilitated compression below 1%. In the present study, only a compliant plate was used, and animals were provided unrestricted access to running wheels with or without resistance. Both the prior and present studies began rehabilitation at 1- week post injury. We found that resistance rehabilitation facilitated defect compression in the ~1–6% range at 4 weeks post injury while rehabilitation without resistance facilitated defect compression in the ~0–2% range. Thus, compression in the range of ~1–5% strain at 3–4 weeks in the course of healing was associated with greater bone formation in both the previous and current studies. Notably, increased rehabilitation intensity was necessary to achieve a similar range of compression in this study. This was likely due to differences in defect geometry and severity of trauma. Researchers have also previously proposed that maintaining a 2–10% strain between fracture ends enables relatively stable secondary fracture healing, while excessive or insufficient strain may affect secondary healing and lead to non-union or delayed union^54,63–67^. However, these observations were made without implantable sensors to track local biomechanics longitudinally, and they relied on various animal models, defect sizes, and fixation strategies. Nevertheless, we found that defect compressive strains for both no resistance and resistance animals were on the higher end of this range after 1 week of rehabilitation. These data demonstrate the importance and potential of patient-specific pre-surgical planning and rehabilitation protocols based on the condition of each injury^68,69^.

In this study, several limitations are worth noting including (1) only female animals were included (2) resistance varied with each revolution during wheel running (3) not all groups were included in each study, and (4) simplification were made for computational models of tissue material behavior. First, female rats were selected to avoid confounding complications due to the rapid skeletal growth and weight gain observed in male rodents. Future work is warranted to investigate the effect of sex on rehabilitation-induced bone regeneration. Second, the resistance brakes exhibited variable resistance throughout each revolution due to brake pad pressure on a spinning wheel that was not perfectly true. The inconsistent resistance level was not fully quantified, but the increased strain on the defect was measured. Third, due to the novel technology that this work involved, we performed a pilot study followed by a larger study to mitigate the chances of unwarranted pain or suffering of rodents. When bone volume and mechanical testing data were compared between studies, the results were statistically equal (Supplemental Fig. S8), thus allowing us to combine results to investigate statistical differences between experimental groups. In addition, all rodents were the same strain, sex, and age, and they all underwent similar orthopedic procedures. A final limitation of the study is the choice of linear isotropic elastic solids for computational modeling of tissue material behavior. This simplification neglects the viscoelastic and fluid properties of healing bone. Nonetheless, this assumption is appropriate for the purpose of quantifying the average ambulatory load transfer to the regenerative niche. Bone growth and remodeling occur on a much larger time scale than ambulatory loads (weeks-months vs. seconds). Previous computational models of tissue differentiation have found that tissue growth kinetics are primarily affected by the transient (i.e., steady state, time-averaged) stress rather than the dynamic stress^70,71^. Thus, we chose not to model viscoelasticity or fluid motion for this study.

Overall, our findings support early resistance running as a post injury treatment for near critically-sized segmental bone defects because it facilitated advantageous loading conditions, which resulted in improved bone healing. Previous literature is contradictory on whether early ambulatory loads are advantageous or deleterious to healing, highlighting the need for personalized measurements to inform rehabilitation decisions that accommodates for fracture type, gemotery, and severity^16,25,28,58^. We combined implantable strain sensors and FEA to highlight a promising platform for tracking patient-specific, real-time defect strains throughout rehabilitation and healing. Future efforts will take advantage of this platform to identify rehabilitation revisions in real-time during rehabilitation in hopes of improving and accelerating functional recovery after traumatic bone injury.

## Figures and Tables

**Fig. 1.** Early resistance rehabilitation improved bone healing. (a) Rehabilitation implemented commercial running wheels (Scurry Rat Running Wheel, Lafayette Instruments^®^) equipped with counters and programmable breaks that can apply resistance and track individual subject’s running activity (number of rotations or distance). Resistance brake pads were modified in-house to apply more consistent friction along the lateral edge of the running wheel (b) Bone volume within centered 1.5 mm of 2 mm defects after 8 weeks of recovery. * p = 0.0306, ordinary one-way Anova with Tukey’s multiple comparisons test. Mean ± standard error. (c-d) *In vivo* representative radiographs and microCT reconstructions reveal early callus formation and consolidation for resistance rehabilitation group only. Scale bar is 1 mm. Triangles = pilot study, squares = follow up study, and circles = animals with sensors implantation from the follow up study. No statistical difference is seen across studies for bone volume results.

**Fig. 2.** Representative histological analysis of regenerated bone at week 8. (a) H&E (b) Goldner’s Trichrome (c) Picrosirius Red (d) Safranin O.

**Fig. 3.** Resistance rehabilitation led to improved functional recovery of femurs with mechanical properties that matched intact femurs. (a) Failure torque of ex vivo femurs under 3°/sec ramp, * p = 0.030628 for sedentary intact versus resistance intact and * p = 0.030628 for sedentary defect versus resistance defect, multiple unpaired t-tests. Mean ± standard error. (b) Torsional stiffness was assessed as the slope of the linear region of the torque-rotation curve. * p = 0.018276 for sedentary intact versus resistance intact and * p= 0.018276 for sedentary defect versus resistance defect, multiple unpaired t-tests. Mean ± standard error. (c) *Ex vivo* polar moment of inertia calculated over 3 mm mid-diaphysis region depicted in microCT reconstructions cross-section. * p = 0.0213, two-tailed unpaired t-test. Mean ± standard error. Triangles = pilot study, squares = follow up study, and circles = animals with sensors implantation from the follow up study. No statistical difference is seen between surgical conditions.

**Fig. 4.** Resistance rehabilitation did not impact weekly activity levels but may improve limb functional restoration. (a) Weekly activity averaged across animals throughout all weeks of activity revealed no significant difference between rehabilitation groups, multiple unpaired t-tests. (b) Longitudinal von Frey demonstrates the impact of injury and subsequent healing on hindlimb mechanical allodynia. Week 0 denotes baseline values for rodents undergoing surgery. Significant effects of time p = 0.0006 and combinatorial time and rehabilitation condition p = 0.0350, mixed effects. # denotes significant effects between timepoints, p=0.0018; * denotes significant effects between groups at week 8, p = 0.0224, mixed effects with Tukey’s multiple comparisons test. Error bars represent standard errors for all graphs.

**Fig. 5.** Rehabilitation intensity modulates defect-level strain distributions. (a) Finite element meshes used to evaluate transfer of loads during ambulation between the fixation plates and defects for individual animals. (b) Transverse plane slices show the spatial distribution of the Young’s modulus for the week 4 models. The no resistance defect displayed intermediate tissue differentiation throughout while the resistance defect only displayed tissue maturation near the intact femur. (c) Longitudinal graph of the fixation plate strain as a function of rehabilitation time. The strain decreased as healing progressed, but resistance was associated with higher strains across all weeks. Mean ± standard error. (d-e) 1 week post injury results. (d) Finite element predictions of 3^rd^ principal (compressive) strain for defect regions are presented in the transverse plane with transparent renderings and slice plots. Strains at 1 week of post injury rehabilitation formed a diagonal band between the proximal-medial and distal-lateral regions of the defect. Resistance led to an increase by a factor of 2.0 in the average compressive strain. (e) Resistance rehabilitation resulted in significantly higher predictions of mean compressive strain within the defect at week 1. ***: p < 0.001, Mann-Whitney U test. Error bars represent 1.5 × inter-quartile range (IQR). (f-g) 4 week post injury results. (f) Finite element predictions of 3^rd^ principal strain at 4 weeks post injury. Strain is concentrated on the lateral and central regions of the defects. Resistance led to a greater than 4-fold increase in the average strain. (g) Resistance rehabilitation resulted in significantly higher predictions of mean compressive strain within the defect at week 4. ***: p < 0.001, Mann-Whitney U test. Error bars represent 1.5 × IQR.

**Table 1.** Summary of material properties used in the model and defect mechanics throughout healing from strain sensors and FE simulations.

Time	Rehabilitation Protocol	Modulus (MPa)	Poisson’s Ratio	Plate Compression (μstrain)	Defect Compressive Strain (%)	Defect Shear Strain (%)	Defect/Plate Strain Ratio
1 wk	No-Resistance	1.0	0.17	1,088 ± 54	−8.9 ± 2.2	5.4 ± 1.3	81.8
1 wk	Resistance	1.0	0.17	2,422 ± 345	−17.8 ± 4.9	11.0 ± 2.9	73.5
4 wk	No-Resistance	134.0 ± 83.7	0.22 ± 0.03	228 ± 25	−0.22 ± 0.19	0.15 ± 0.11	9.7
4 wk	Resistance	49.4 ± 48.2	0.19 ± 0.02	754 ± 61	−0.98 ± 0.72	0.68 ± 0.40	13.0

## Data Availability

The authors declare that the data supporting the findings of this study are available within the article and its Supplementary Figures. The dataset supporting the findings of this study are also available from the corresponding author upon request.
